# Levels of Leydig cell autophagy regulate the fertility of male naked mole-rats

**DOI:** 10.18632/oncotarget.22088

**Published:** 2017-10-26

**Authors:** Wenjing Yang, Li Li, Xiaofeng Huang, Guanghan Kan, Lifang Lin, Jishuai Cheng, Chen Xu, Wei Sun, Wei Cong, Shanmin Zhao, Shufang Cui

**Affiliations:** ^1^ Laboratory Animal Center, Second Military Medical University, Shanghai, China; ^2^ Department of Training, Second Military Medical University, Shanghai, China; ^3^ Medical Record Department, Ministry of Information, Changzheng Hospital, Second Military Medical University, Shanghai, China; ^4^ China Astronaut Research and Training Center, Beijing, China

**Keywords:** naked mole-rats, autophagy, leydig cells, testis, nonbreeding

## Abstract

Fertility is abolished in nonbreeding males in colonies of natal naked mole-rats (NMRs). Although spermatogenesis occurs in both breeding and nonbreeding male NMRs, the mechanisms underlying the differences in fertility between breeders and nonbreeders remain unexplored. In this study, a significant decrease in autophagy was observed in Leydig cells of the testis from nonbreeding male NMRs. This alteration was visualised as a significant decrease in the levels of autophagy-related gene 7 (Atg7), Atg5, microtubule-associated protein 1A/B light chain 3 (LC3-II/I) and the number of autophagosomes and an increase in P62 levels using Western blotting analyses. Furthermore, monodansylcadaverine (MDC) staining and Western blot analyses revealed that testosterone production decreased in nonbreeding male NMR Leydig cells, this decrease was associated with a reduction in autophagy. Primary Leydig cells from breeding and nonbreeding male NMRs were processed to investigate the effect of an autophagy inhibitor (3-MA, 3-methyladenine) or an autophagy activator (rapamycin) on testosterone production. Rapamycin induced an increase in testosterone production in NMR Leydig cells, whereas 3-MA had the opposite effect. Consequently, spermatogenesis, the weight of the testis, and androgen levels were dramatically reduced in nonbreeding male NMRs. While rapamycin treatment restored the fertility of nonbreeding male NMRs. Based on these results, inadequate autophagy correlates with a decrease in steroid production in nonbreeding male NMR Leydig cells, which may ultimately influence the spermatogenesis and fertilities of these animals.

## INTRODUCTION

The naked mole-rat (*Heterocephalus glaber*; NMR) is a highly eusocial rodent that inhabits East Africa. Colonies often consist of 40–80 individuals and may contain up to 300 individuals [1, 2]. Only one breeding female (the queen) and 1–3 breeding males are present in an NMR colony. The other colony members are either sterile or do not reproduce for other reasons, such as behavioural inhibition [3, 4]. Fertility has been shown to be suppressed in nonbreeding, mature female NMRs, but this suppression is reversible [5]. Suppressed females share similarities with juvenile mammals of other species [6] and mammals in which reproductive activity is suspended during seasonal anestrus [7, 8]. According to the study by Faulkes et al. [9], plasma luteinising hormone (LH) levels are lower in nonbreeding male NMRs than in breeding males, and the fertility of nonbreeding males is rescued by the administration of gonadotropin-releasing hormone (GnRH). However, some nonbreeding males have active sperm [5]. The hypothalamic–pituitary gonadal axis plays critical roles in male reproductive organs and the endocrine system controls the secretion of androgens and spermatogenesis. However, further studies are required to determine whether pathways other than the male reproductive organ regulate the fertility of male NMRs.

Mammalian spermatogenesis is a highly complex process that consists of cell division and differentiation. Spermatogonia undergo several cycles of mitosis followed by meiosis, resulting in spermatocyte production [10]. During spermiogenesis, spermatocytes form spermatids in the seminiferous epithelium, which are subsequently released into the lumen [10]. A series of changes occur during spermatogenesis that are associated with the differentiation of haploid round spermatids to spermatozoa. Murine spermiogenesis is divided into the following four phases based on sharp nuclear condensation: round spermatids, elongating spermatids, condensing spermatids and condensed spermatids [11]. For many decades, Leydig cells have been known to produce testosterone to support spermatogenesis [12, 13]. Testosterone levels in the adult testis are maintained at a relatively stable and high level (200–800 nM) [10].The testosterone levels and Leydig cell functions in NMRs remain largely unexplored.

Autophagy is a primary intracellular catabolic process that controls cell homeostasis by degrading long-lived proteins and recycling self-cytosolic components [14–17]. Autophagy is characterised by a double-membrane vesicle called an autophagosome that contains cytoplasmic materials [18–21]. Autophagosomes move along cytoskeletal structures and fuse with lysosomes to form autolysosomes, in which both the autophagosome membrane and its contents are degraded by resident hydrolases [22–25]. Autophagy is evolutionarily conserved in all eukaryotic organisms [22] and is involved in many physiological processes in mammalian systems, including cell growth, survival, differentiation, and development [26–28]. Dysfunctional autophagy has also been shown to participate in pathological conditions [14, 24, 26–34]. Autophagy-related gene 12 (Atg12)/Atg5 and microtubule protein 1A/B light chain 3 (LC3)-conjugated lipids/membranes are two ubiquitin-like conjugation systems that critically regulate the formation of autophagosomes. Both Atg12 and Atg5 are activated by Atg7, which is homologous to the ubiquitin-activating enzyme E1 (Uba1) gene encoding an E1-like enzyme that is critical for these conjugation systems [22] and required for the induction of both selective and nonselective autophagy [23, 25]. Autophagy plays an essential role in regulating both testicular development [35] and spermatogenesis during adulthood [18].

The circulating androgen concentration in nonbreeding males is adequate for spermatogenesis [9]. However, researchers have not determined whether autophagy participates in regulating androgen synthesis, NMR Leydig cell functions and spermatogenesis. Additionally, further investigations are required to determine whether differences in the structures of the reproductive system, particularly the testis and epididymis, or the specific processes involved in spermatogenesis are responsible for regulating reproductive functions.

## RESULTS

### Differences in testicular histopathology and morphometry between breeding and nonbreeding male NMRs

**Figure 1:**
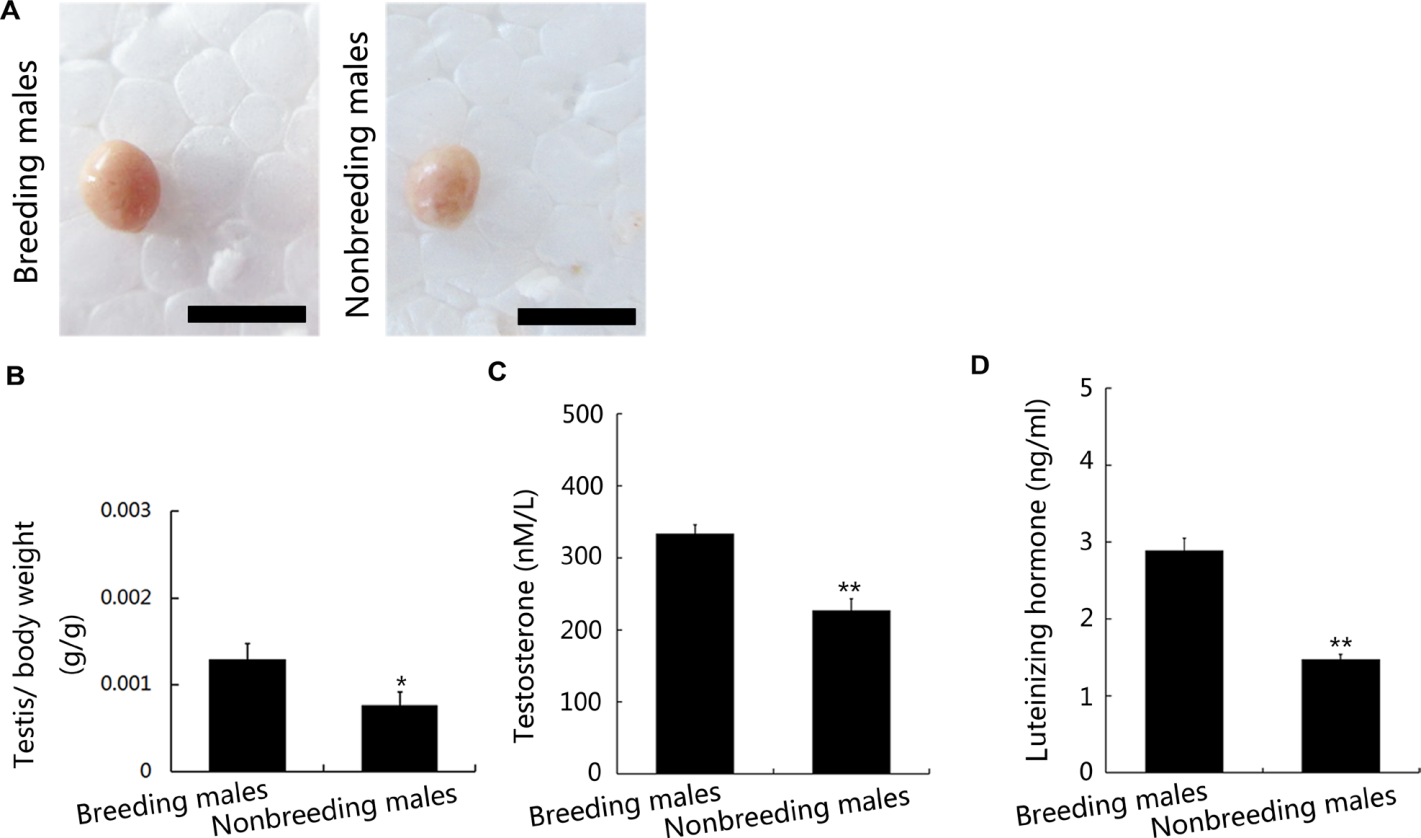
Testes weights and androgen levels in breeding and nonbreeding male NMRs (**A**) The testes of breeding and nonbreeding male NMRs (*P* = 0.03694). (**B**) Testes weights of breeding and nonbreeding male NMRs. (**C** and **D**) Plasma testosterone (*P* = 0.001849) and LH levels (*P* = 0.0002026, *n* = 10 in each group) were quantified using enzyme-linked immunosorbent assays (ELISAs). Scale bars = 5 mm. Data are presented as means ± SEM.

**Figure 2:**
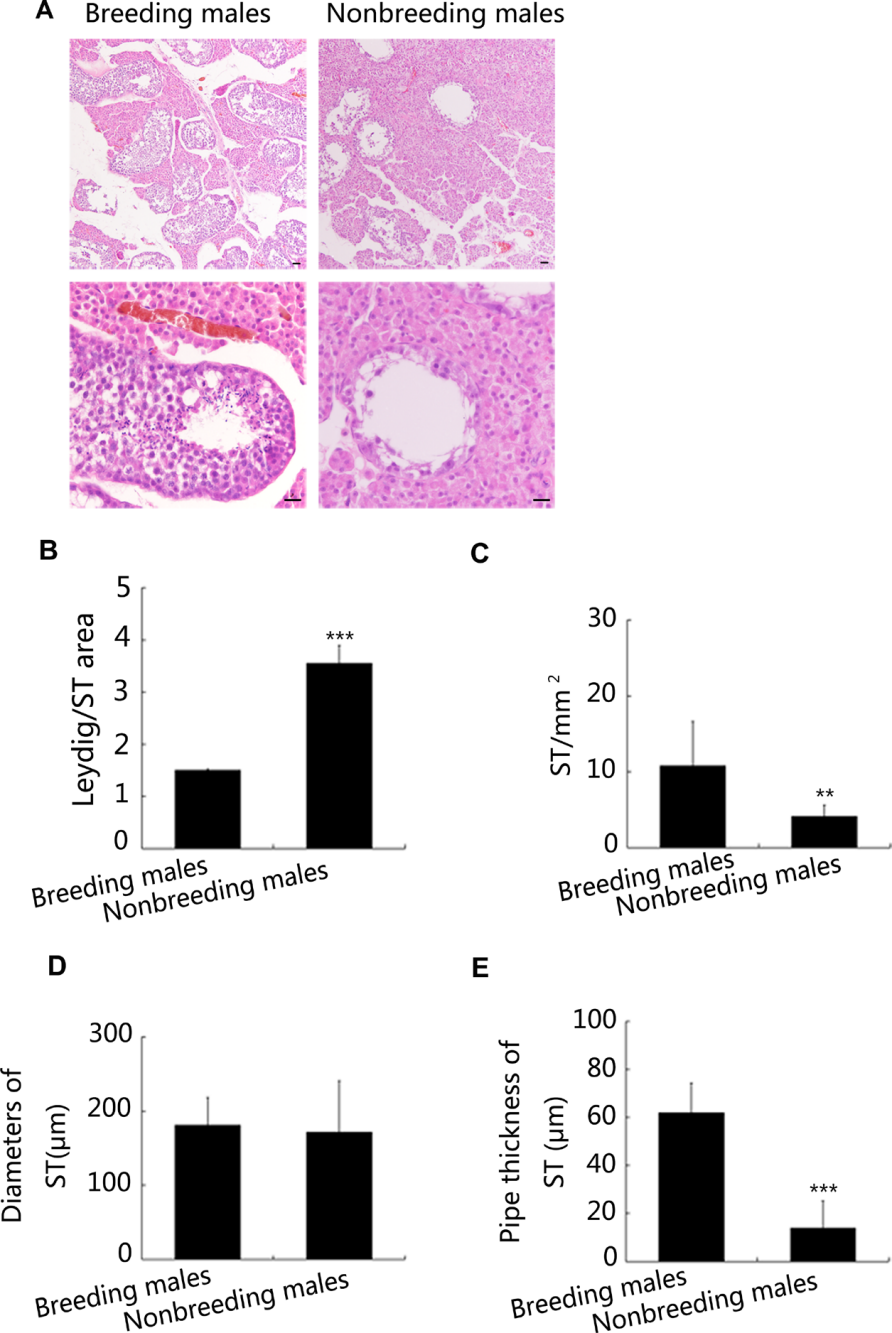
Severe reproductive defects in nonbreeding male NMRs (**A**) Histology of the Leydig cell areas and STs of breeding and nonbreeding male NMRs. (**B**–**E**) Quantification of the Leydig cell area/ST area ratio (*P* = 0.0006670), ST areas (*P* = 0.003640), ST thickness (*P* = 0.0007127), and ST diameters (*P* = 0.003640) (*n* = 6 in each group). Scale bars = 20 μm. Data are presented as means ± SEM.

The weights of the testes from breeding and nonbreeding male NMRs were evaluated. The weights of the testes from nonbreeding male NMRs were significantly lower than the weights of the testes obtained from breeding male NMRs with similar body weights (Figure 1A and 1B). Consistent with these results, plasma testosterone and LH levels were significantly lower in the nonbreeding male NMRs *in vivo* (Figure 1C). Based on the results of the histological analyses, unlike other rodents [18], NMR testes are characterised by a relatively large area in which Leydig cells are distributed (Figure 2A and 2B). Furthermore, the density of seminiferous tubules in the testes was much higher in breeding male NMRs than in nonbreeding males (Figure 2A and 2C). Although the diameter of the testicular seminiferous tubules was comparable between nonbreeding and breeding male NMRs (Figure 2A and 2D), the thickness of the seminiferous tubules was much greater in breeding males than in nonbreeding males (Figure 2A and 2E).

**Figure 3:**
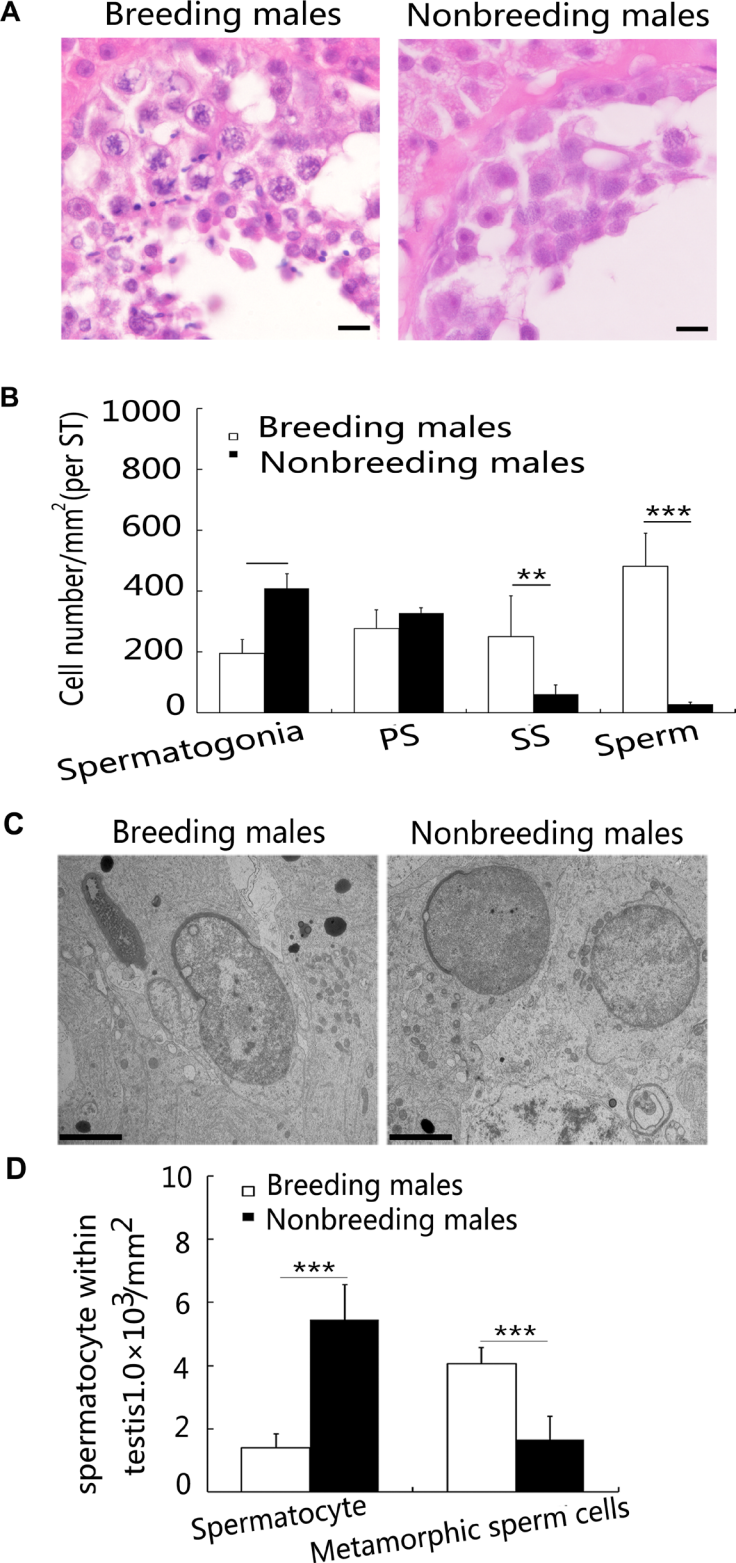
Differences in spermatogenesis between breeding and nonbreeding male NMRs (**A**) Histology of the ST in breeding and nonbreeding male NMRs. (**B**) Quantification of the numbers of spermatogonia (*P* = 0.004952), primary spermatocytes (PS) (*P* = 0.2378), secondary spermatocytes (SS) (*P* = 0.003477), and sperm in ST (*P* = 0.002243). Scale bars = 20 μm. (**C**) TEM was used to investigate spermatogenesis in breeding and nonbreeding male NMR testes (spermatocytes, *P* = 0.0001460, metamorphic sperm cells, *P* = 0.0006650) (*n* = 6 in each group). Scale bars = 2 μm. (**D**) Quantification of the numbers of spermatids at different maturation stages. Data are presented as means ± SEM.

Investigations of different stages of sperm cell development within the testicular seminiferous tubule epithelium revealed greater numbers of mature sperm and a relative increase in the numbers of secondary spermatocytes in breeding males. However, fewer spermatogonia were observed in the testicular seminiferous tubule basilar membrane of breeding males (Figure 3A and 3B). Based on the transmission electron microscopy (TEM) results, a greater number sperm cells was in the metamorphic stage in the testis of breeding male NMRs, but a greater proportion of sperm cells was in the spermatocyte stage in nonbreeding male NMRs (Figure 3C and 3D). Although macrophages are located along tubules and affect the ability of undifferentiated spermatogonia to differentiate and proceed through spermatogenesis, only the numbers of spermatogonia and not macrophages were calculated. These results are consistent with previous findings reported by Faulkes et al. [9, 36].

### Sperm density is lower in the epididymis in nonbreeding male NMRs than in breeding male NMRs

**Figure 4:**
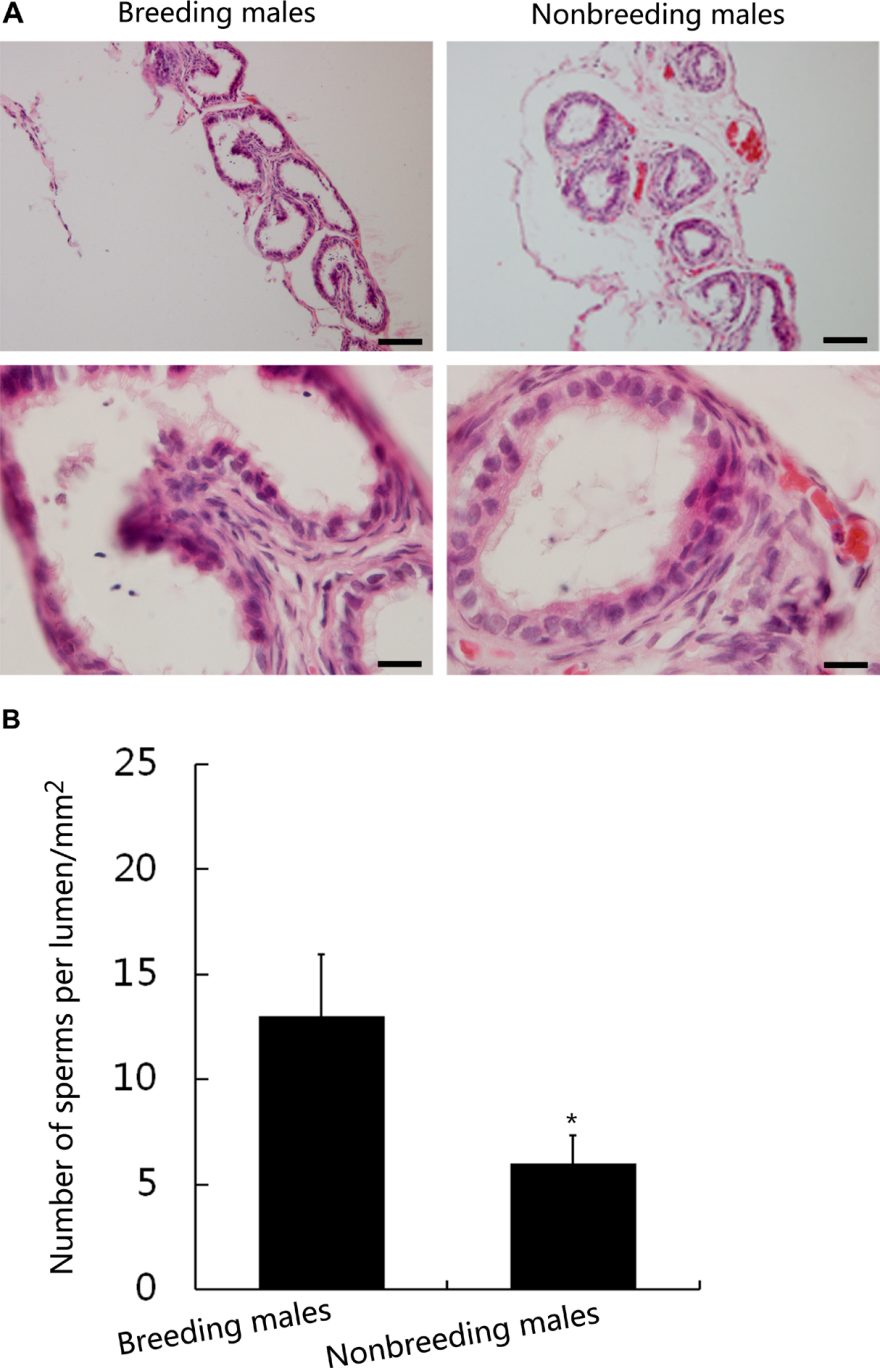
Sperm distribution in the cauda epididymis of breeding and nonbreeding male NMRs (**A**) Histology of the cauda epididymis in breeding and nonbreeding male NMRs. (**B**) The total number of sperm in the cauda epididymis of breeding and nonbreeding male NMRs (*P* = 0.03805, *n* = 6 in each group). Scale bars = 20 μm. Data are presented as means ± SEM.

We did not observe a difference in the diameter of the epididymis cavity between breeding and nonbreeding male NMRs. Several sperm were observed in both breeding and nonbreeding male NMRs (Figure 4A), but fewer sperm were observed within the epididymis cavity in nonbreeding male NMRs (Figure 4B). Thus, the sperm storage functions of NMRs, particularly nonbreeding NMRs, are highly inferior to other rodents, such as mice [18].

### Decreased autophagy levels in nonbreeding male NMRs

**Figure 5:**
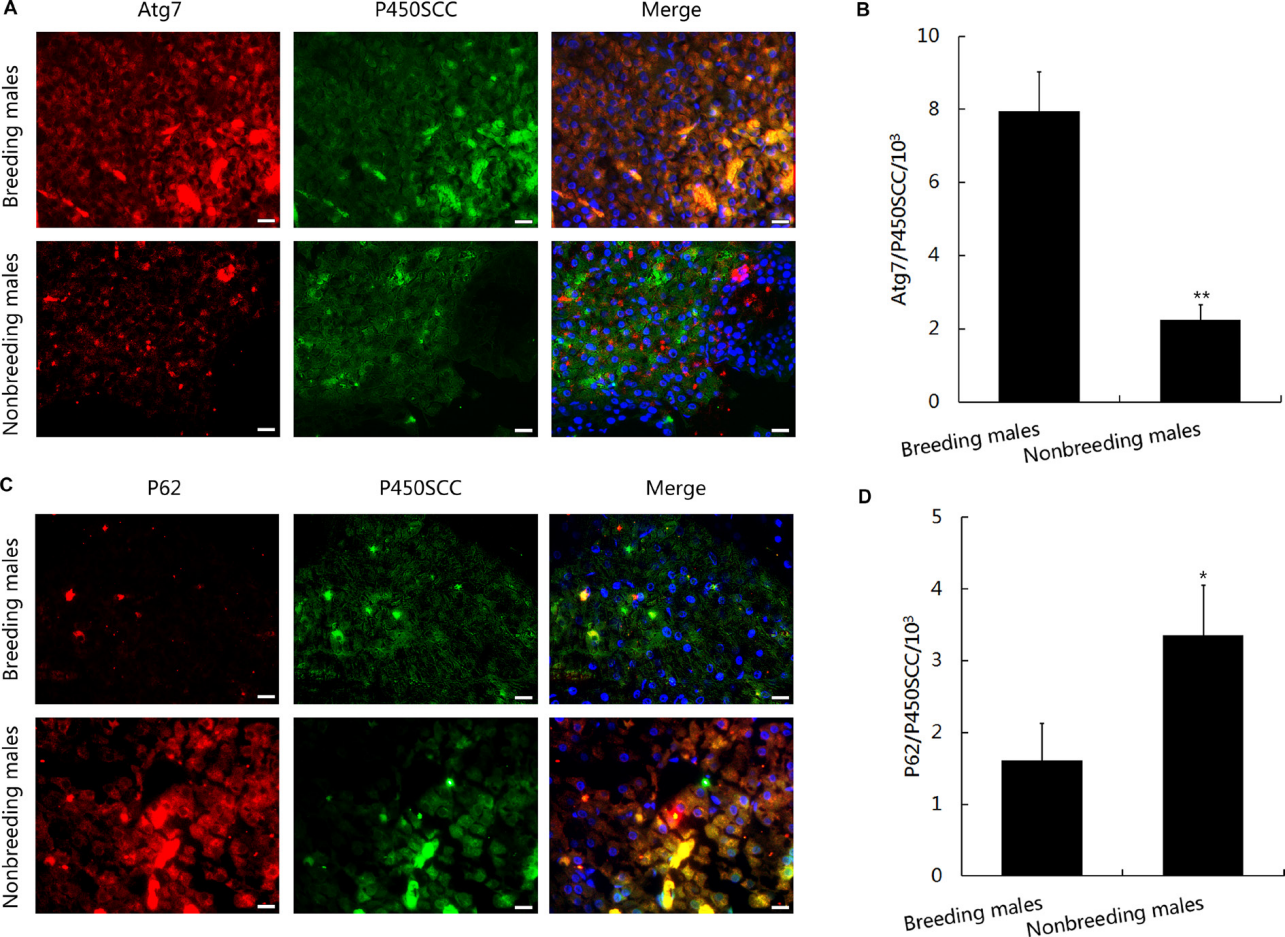
Immunohistochemical detection of autophagy levels in leydig cells within breeding and nonbreeding male NMR testes (**A** and **B**) Double-immunostaining for P450SCC (markers for Leydig cells) with Atg7 or P62 in breeding and nonbreeding male NMR testes. (**C** and **D**) Densities of Atg7^+^/P450SCC^+^ cells (*P* = 0.002155) and P62^+^/P450SCC^+^ cells (*P* = 0.04640) in breeding and nonbreeding male NMR Leydig cell areas (*n* = 6 in each group). Scale bars = 20 μm. Data are presented as means ± SEM.

**Figure 6:**
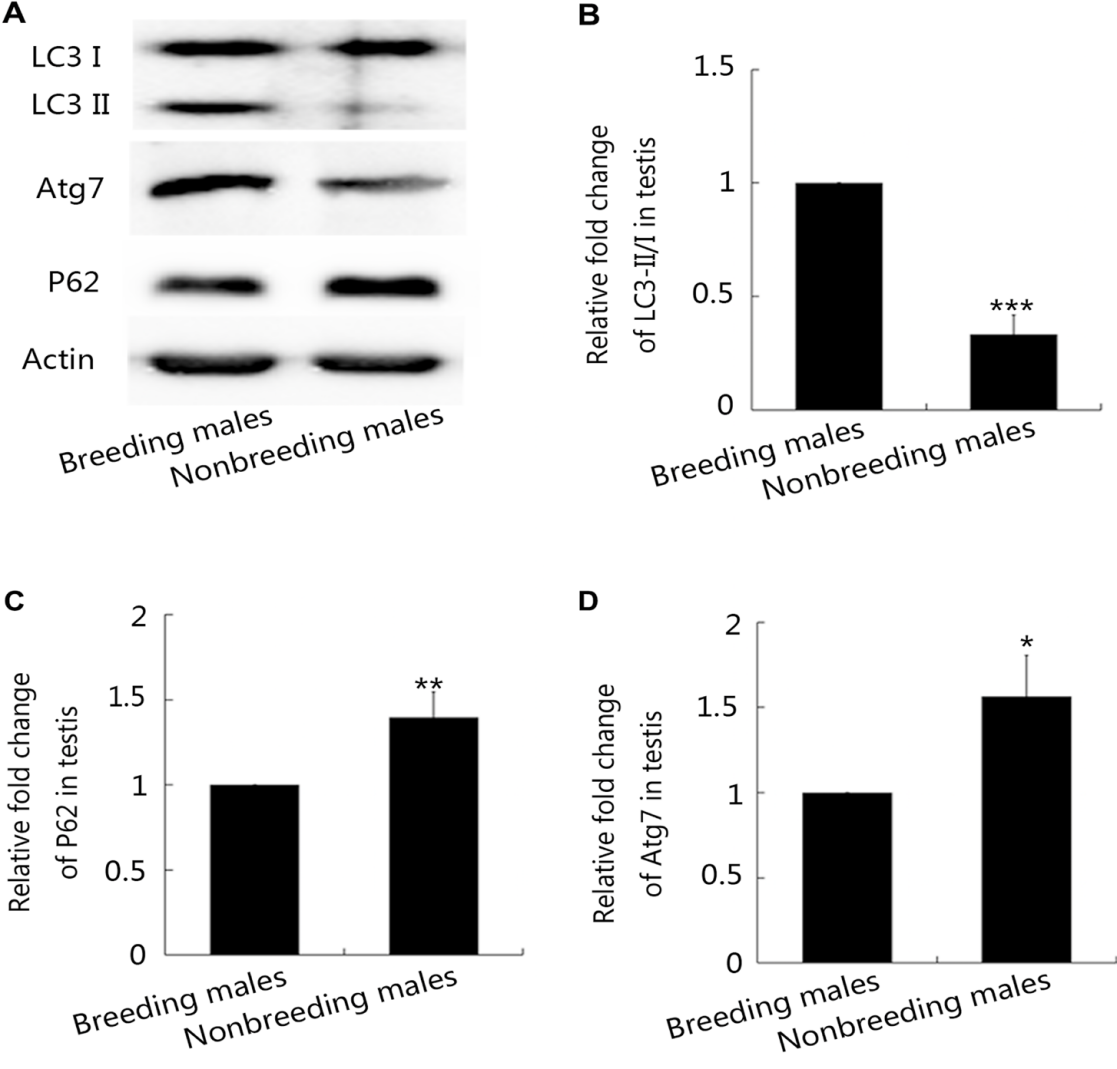
Western blot analysis of autophagy levels in breeding and nonbreeding male NMR testes (**A**) Western blot analysis of LC3-I, LC3-II, Atg7 and P62 levels in breeding and nonbreeding male NMR testes. (**B**–**D**) Quantification of LC3-I/LC3-II (*P* = 0.01006), Atg7 (*P* = 0.004855) and P62 (*P* = 0.004233) levels in breeding and nonbreeding male NMR testes (*n* = 6 in each group). Data are presented as means ± SEM.

Because Atg7 is an E1-like enzyme that is required to induce autophagy, we first evaluated Atg7 levels in NMRs. Based on the immunohistochemical staining and Western blot analysis, Atg7 levels were dramatically increased in the Leydig cells from breeding males compared with nonbreeding males (Figures 5A, 5C, 6A, and 6D). In addition, levels of the autophagy substrate SQSTM1/p62 were higher in the immunofluorescence staining (Figures 5B, 5D, 6A, and 6C), indicating that protein degradation was induced in NMRs. Lower Atg7 levels were observed in nonbreeding male NMRs, resulting in the reduced consumption of P62 (Figure 6A and 6D). Meanwhile, the levels of Atg5, which is also required for mammalian autophagy [37], were dramatically decreased in nonbreeding males compared with breeding males (Supplementary Figure 1). Atg7 also functions as an E1-like enzyme by activating two Ubl conjugation systems (LC3 and Atg12) and conjugating LC3 to lipids/membranes during spermatogenesis. Conjugated LC3 might help the proacrosomal vesicle to fuse and expand, similar to its function in autolysosome formation [40–42]. We therefore examined LC3 expression using Western blot analyses. The ratio of LC3-II/LC3-I was much higher in breeding male NMRs (Figure 6A and 6B), indicating that these males exhibited a higher level of autophagy.

### Deficient autophagy in the Leydig cells of nonbreeding male NMR testes is associated with spermatogenesis

**Figure 7:**
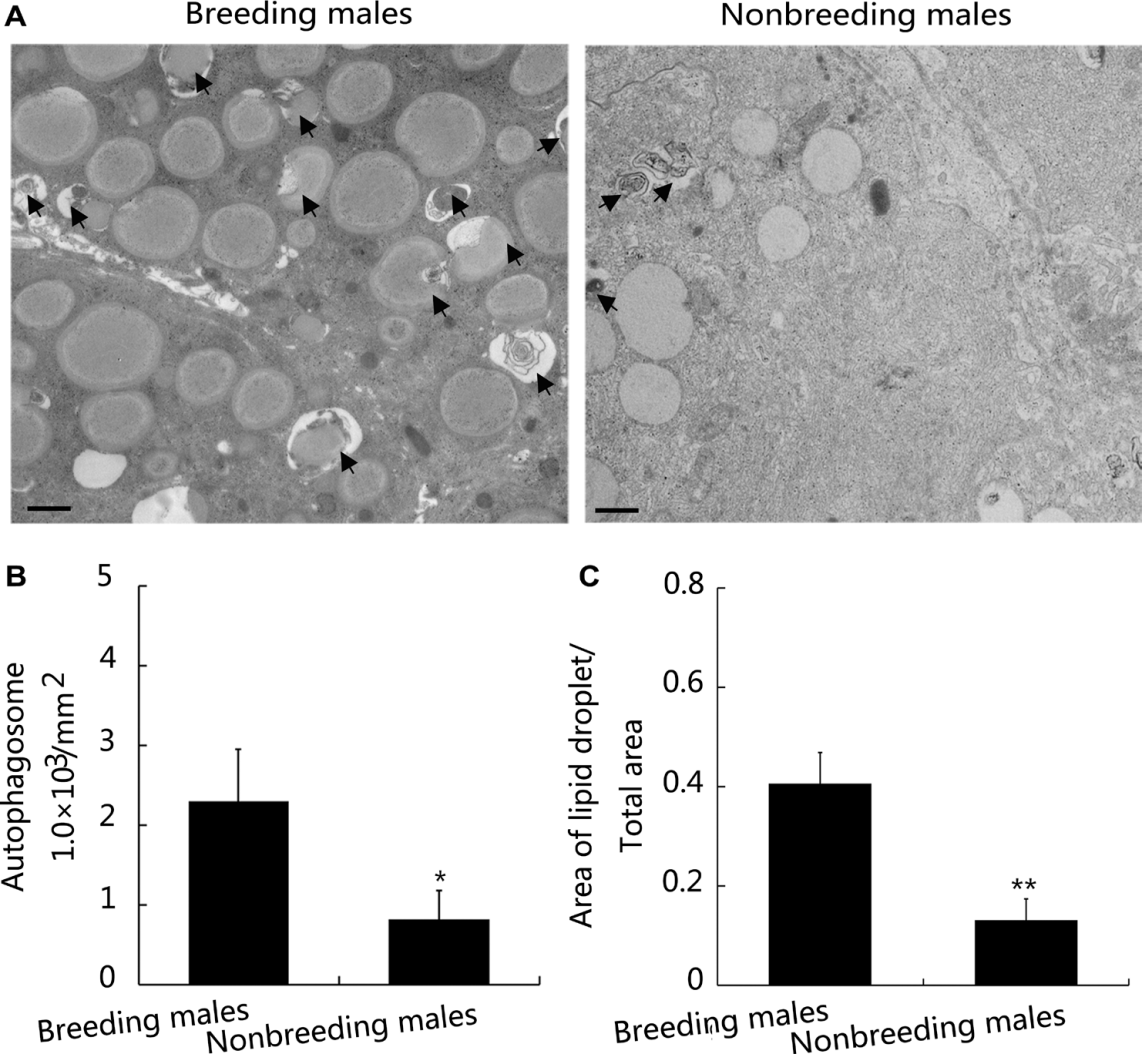
Autophagy levels in Leydig cells and analysis of spermatogenesis in breeding and nonbreeding male NMR testes (**A**) TEM was used to visualise Leydig cells from breeding and nonbreeding male NMRs. Scale bars = 1 μm. (**B** and **C**) Quantification of the number of autophagosomes (arrows) (*P* = 0.04846) and lipid droplets (*P* = 0.006703) shown in (A) (*n* = 6 in each group). Data are presented as means ± SEM.

Because we observed high levels of autophagy in Leydig cells from NMRs, we used TEM to observe the ultrastructural anatomy of autophagosomes in Leydig cells *in vivo*. A much higher density of autophagosomes was observed in the Leydig cells of testis from breeding males than in nonbreeding male NMRs (Figure 7A and 7B). Moreover, in breeding male NMR Leydig cells, the area containing lipid droplets was much larger than the area that excluded lipid droplets (Figure 7A and 7C). When the nonbreeding male NMRs were treated with rapamycin, both the autophagosomes and the levels of testosterone and LH were dramatically increased (Supplementary Figure 2A–2D). HE staining showed that after the treatment of rapamycin, both numbers of mature sperms and testis weights in nonbreeding males increased significantly compared with breeding males (Supplementary Figure 2E–2F) Based on these results, the high level of autophagy observed in the nonbreeding male NMR testis may be associated with hysteretic spermatogenesis and is potentially responsible for the observed alterations in androgen synthesis, which blocked sperm maturation.

### Alterations in autophagy activity affect testosterone production in primary NMR Leydig cells *in vitro*

**Figure 8:**
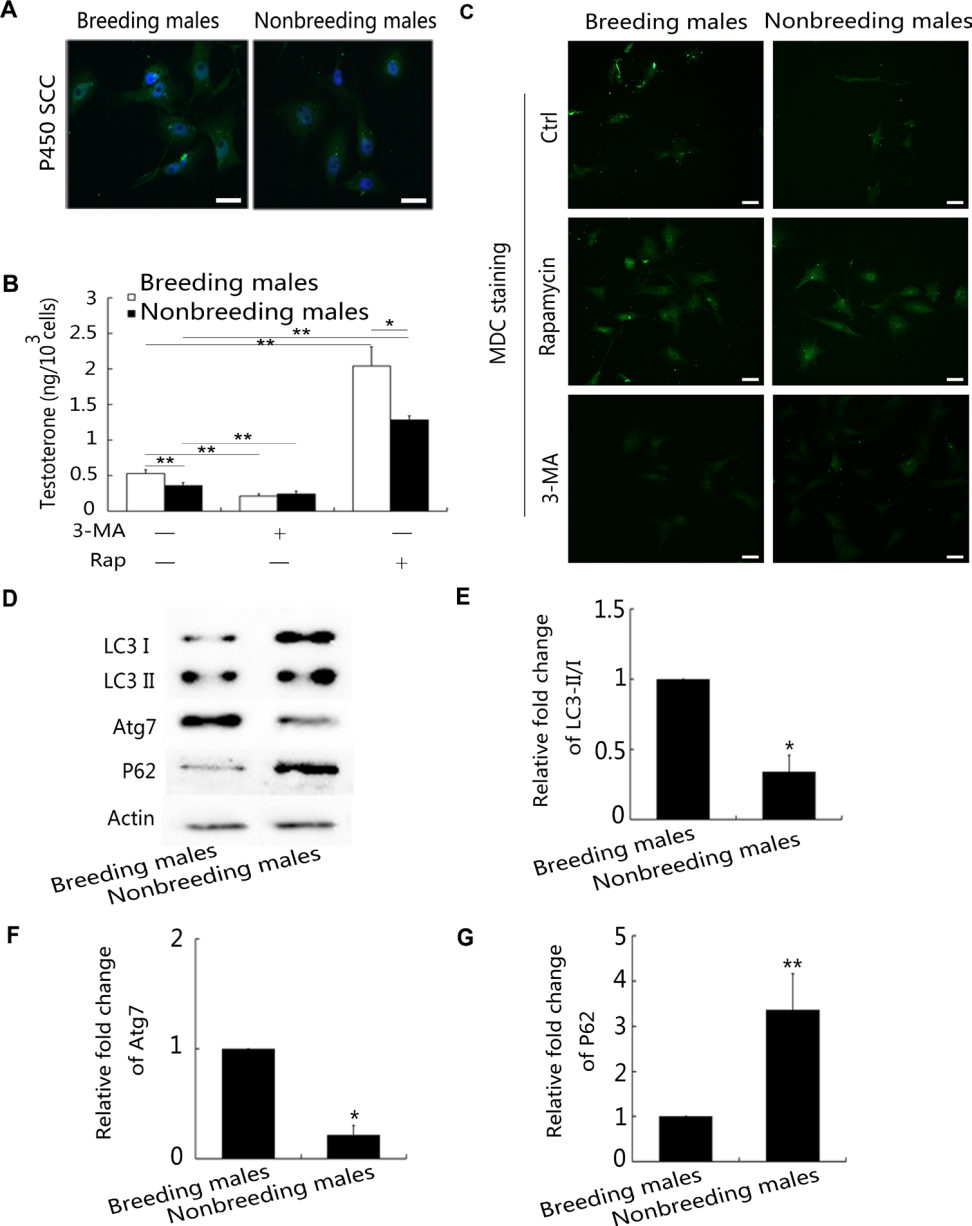
High autophagy levels in Leydig cells inhibit testosterone production (**A**) Identification of Leydig cells in NMRs using anti-P450SCC antibodies. (**B**) Testosterone concentrations in Leydig cells obtained from breeding and nonbreeding male NMRs were measured using radioimmunoassays. NMRs were treated with 3-MA or rapamycin. (**C**) MDC staining in Leydig cells obtained from breeding and nonbreeding male NMRs. (**D**) Western blot analyses were used to determine the levels of the LC3-I, LC3-II, Atg7, and P62 proteins in Leydig cells obtained from breeding and nonbreeding male NMRs. (**E–G**) Quantification of LC3-I/LC3-II (*P* = 0.001063), Atg7 (*P* = 0.001341), and P62 (*P* = 0.009624) levels in the Leydig cells from breeding and nonbreeding male NMRs (*n* = 4 in each group). Data are presented as means ± SEM.

Leydig cells are located in the testicle and produce testosterone. They are present in clusters in the interstitial spaces between seminiferous tubules in the testes, and more than 700 million Leydig cells have been observed [38, 39]. An increase in testosterone levels induces the development of secondary sexual characteristics and sexual reproduction [21]. In this study, we first detected the level of autophagy in breeding and nonbreeding male NMR Leydig cells using ani-P450SCC [40] antibodies (Figure 8A). Much higher levels of testosterone were synthesised by Leydig cells in breeding than in nonbreeding male NMRs, according to the results of radioimmunoassays (Figure 8B). Based on the results of the monodansylcadaverine (MDC) staining, more autophagosomes were observed in the Leydig cells of nonbreeding male NMRs than breeding male NMRs (Figure 8C). Primary Leydig cells obtained from breeding and nonbreeding male NMRs were used to investigate how changes in the level of autophagy, which were experimentally induced using an autophagy inhibitor (3-MA) or activator (rapamycin), affected Leydig cell testosterone production. The addition of rapamycin resulted in an increase in testosterone production in both types of NMR Leydig cells, and the opposite effect was observed when Leydig cells were treated with 3-MA (Figure 8C). Finally, according to the results of the Western blot analysis, the LC3-II/LC3-I ratio was significantly higher in nonbreeding male NMR (Figure 8D–8G).

## DISCUSSION

The testes of nonbreeding male NMR are much smaller than the testes of breeding males, as measured using both the absolute weight and weight relative to their body mass. Lower plasma LH and testosterone concentrations might account for the insufficient growth observed in nonbreeding male NMRs, consistent with the results from previous studies [9]. The suppressed endocrine levels in nonbreeding male NMRs may result in an elimination of mating choice by the oestrual queen or the suppression of their reproductive function [9]. The key question to answer is how these differences in physiology between breeding and nonbreeding male NMRs result in differences in fertility when spermatogenesis is maintained in both types of males.

According to the results of histological examinations, both nonbreeding and breeding male NMRs possess a large number of Leydig cells and sparsely distributed seminiferous tubules (STs); fewer STs were observed in breeding male NMRs. Leydig cells comprise approximately 60% of the testicular mass [41]. One of the most critical functions of Leydig cells, which are distributed in the interstitial tissue of the testis, is to synthesise and secrete testosterone and LH [38, 39], which regulate spermatogenesis. Macrophages are located along the tubules and regulate the ability of undifferentiated spermatogonia to differentiate and proceed through spermatogenesis, but only spermatogonia and other sperm cells at different stages were calculated for investigations on the structures of STs. Based on the results of histological examinations, the density of Leydig cells was much higher in breeding males than in nonbreeding male NMRs. However, plasma testosterone and LH levels and the maturation of sperm cells were significantly decreased in nonbreeding male NMR testes. The key issue is to determine how these differences in physiology lead to differences in fertility between breeding and nonbreeding male NMRs, because spermatogenesis is maintained in both types of males. Although spermatogenesis occurred in all males examined, differences were observed in the levels of mature sperm cells in breeding and nonbreeding male NMRs, and thus Leydig cells may have normal functions in regulating sperm cell development. In summary, decreased steroidogenesis is associated with low autophagy levels in nonbreeding male NMR Leydig cells.

Autophagy is a cellular degradation pathway that involves the delivery of cytoplasmic cargo to lysosomes [24, 42–44]. This pathway is required for many processes, including cell survival, differentiation, malignant proliferation, development and homeostasis [24, 42–44]. Based on accumulating evidence, dysfunctional autophagy is associated with diseases, particularly cancer [45, 46]. As shown in the study by Qu et al., mice lacking Beclin-1 tend to develop spontaneous tumours [47], indicating that autophagy may suppress tumourigenesis in established cancers. NMRs are known for their longevity and resistance to cancer. Compared with laboratory rodents and other mammalian species with similar life spans, NMRs obviously have quite a low tumourigenesis rate. To date, Delaney et al. [48] and Taylor et al. [49] have identified two and four cases of spontaneous tumours in NMRs aged from 6 to 24 years old, respectively, including hepatocellular carcinoma and interstitial (Leydig) cell tumours,. Although relatively high levels of autophagy may participate in promoting good health [19, 20], the mechanisms underlying the tumourigenesis observed in the 6 NMRs suffering from spontaneous tumours and their association with autophagy levels in their bodies remain an enigma. Meanwhile, researchers have investigated the specific traits that endow NMRs with the ability to resist tumourigenesis through non-autophagy ways [50, 51]. According to Tian et al., an increase in the levels of high molecular weight hyaluronan secreted by NMR fibroblasts reduces growth due to an increase in the activity of contact inhibition pathways that activate the tumour suppressor locus inhibitor of cyclin-dependent kinases 4a/b [52].

During the initial phase of autophagy, LC3 is activated by Atg7 and then conjugated to lipids/membranes, where it induces membrane expansion and vesicle completion [18, 20, 53]. As shown in the study by Masiero et al., an Atg7 deletion induced muscle atrophy and the accumulation of aberrant mitochondria and swollen sarcoplasmic reticulum [54]. Leydig cells have been shown to exhibit higher levels of autophagy than other non-steroid-producing cells in mice and rats [42, 55], possibly because of the increased cellular demand for autophagy imposed by the higher turnover rate of components of the steroid-producing apparatus. Thus, alterations in autophagy activity may result in inadequate steroid secretion. Indeed, much lower Atg7 and LC3-II/I levels were observed in the Leydig cells of nonbreeding male NMRs and were associated with higher levels of P62, an autophagy substrate, in our study. Additionally, TEM revealed more autophagosomes surrounding the lipid droplets in the Leydig cells of breeding male NMRs, and MDC staining and Western blot analysis showed a higher level of autophagy in the Leydig cells of primary breeding males than in nonbreeding male NMRs. Moreover, based on the results of radioimmunoassays, a significantly lower level of testosterone was produced by the Leydig cells of primary breeding male NMRs, likely representing a side effect of enhanced autophagy on steroidogenesis. TEM investigations of the ST areas of NMR showed that maturation of the sperm cells was significantly inhibited in nonbreeding male NMR testes. We propose that deficient autophagy may be the cause of the decreased steroidogenesis observed in nonbreeding male NMR Leydig cells, and this deficiency may delay the progress of spermatogenesis. We also evaluated testosterone production in breeding and nonbreeding male NMR Leydig cells that were treated with 3-MA and rapamycin to artificially alter autophagy levels. We then tested the reproductive capacities of both types of males *in vivo*.

In conclusion, a deficiency in autophagy decreases the production of testosterone by Leydig cells and suppresses the spermatogenesis in nonbreeding male NMRs, which may result in the infertility of these males.

## MATERIALS AND METHODS

### Animals

Captive colonies of NMRs were maintained at the Institute of Zoology, Second Military Medical University, Shanghai, China. Mole-rats were housed in artificial Perspex burrow systems that consisted of a nest, food and toilet chambers as described by Faulkes et al. [56]. Fourteen male NMRs, including 7 breeders and 7 subordinates, at the age of 18 months with a weight of 45.4 ± 2.3 g were used in this study. The breeders were adult males that regularly consorted (via naso-anal grooming) with the queen and had been observed to copulate with the queen during the oestrus period. Subordinate males were also adults but had never been observed to consort or copulate with the queen.

### Rapamycin injection

NMRs received intraperitoneal (IP) injections of either rapamycin (2 mg/kg per day, IP for 30 days) or vehicle daily [57, 58]. NMRs were weighed before rapamycin administration. Rapamycin and vehicle were administered 1 h before exposure to hypoxia and normoxia. Then, NMRs were perfused or sampled for further investigations following the final rapamycin injection. Rapamycin was dissolved in ethanol at a stock concentration of 25 mg/ml and stored at −20°C. A 1-mg/ml working solution was generated immediately before administration comprising 4% rapamycin or ethanol, 5% Tween-80 (Fisher Biosciences), and 5% PEG400 (Sigma-Aldrich), as previously described [59–61].

### Cell culture

Primary Leydig cells were isolated from 12-month-old breeding and nonbreeding male NMRs according to previously described protocols for mice [40], with some modifications. The testes were decapsulated and washed three times with PBS solution supplemented with 100 UI/ml streptomycin and penicillin to extrude the sperm. The testes were then cut into 1-mm^3^ pieces that were digested with 10 ml of a solution containing Dulbecco's Modified Eagle's Medium/F-12 supplement (1:1 v/v) (DMEM/F-12), 200 μg/ml deoxyribonuclease I, and 0.5 mg/ml collagenase IA. The digested tissues were incubated for 20 min at 33°C in a shaking water bath at a speed of 70 oscillations/min and then layered over 40 ml of 5% Percoll (Gibco) and allowed to settle for 20 min. The top 35 ml of the Percoll (which included the Leydig cells and other interstitial cells that were digested from the intact tubule tissues) were harvested and centrifuged at 500 g for 10 min at 4°C. The pellet was then resuspended in 55% Percoll and centrifuged at 20,000 g for 30 min at 4°C. After the top 2 ml of Percoll (containing cellular debris and epithelial cells) were removed, a 5-ml fraction was collected from the top of the tube. Primary Leydig cells were cultured in Minimal Essential Medium (MEM+GlutaMAX, Gibco) supplemented with 1x penicillin/streptomycin, 25 mM HEPES (pH 7.4) and 0.07% BSA at 33°C in 5% CO_2_. After cells were allowed to attach and starved for 3 h, the cells were washed once with culture medium and then used in experiments. Autophagy was modulated in NMR Leydig cells using 50 nM rapamycin or 20 mM 3-MA.

### Western blot analysis

Tissue extracts were processed using a tissue lyser in cold radioimmunoprecipitation assay (RIPA) buffer (Beyotime Biotechnology, China) supplemented with phenylmethylsulfonyl fluoride (1 mM) and a protease inhibitor cocktail (Roche, Switzerland). The lysates were centrifuged at 12,000 rpm for 15 min at 4°C, and the supernatant was then harvested. A Bio-Rad protein assay was used to quantify protein concentrations. Samples were separated via SDS-PAGE and then electrotransferred to a PVDF membrane. After the membranes were blocked with 10% non-fat dry milk in buffer containing 20 mM Tris-HCl (pH 7.6), 150 mM sodium chloride and 0.1% Tween-20 (TBST) for 2 h at room temperature, the membranes were incubated with rabbit polyclonal antibodies against LC3 (1:1,000 dilution, Proteintech, Chicago, IL), P62 (1:1,000, Proteintech, Chicago, IL), Atg7 (1:1,000, Proteintech, Chicago, IL), and β-actin (1:1,000 dilution, Proteintech, Chicago, IL) overnight at 4°C. The membranes were then incubated with horseradish peroxidase (HRP)-conjugated secondary antibodies (Kangcheng Biotechnology, 1:10,000) diluted in TBST for 1 h at room temperature. The membranes were scanned using a Gel Logic Imaging System (Carestream, USA). Band intensities were quantified using an Image-Pro Plus 6.0 analysis system.

### TEM

Tissues were fixed with saline (PBS, 4°C, 0.1 M) containing 2.5% glutaraldehyde and 4% paraformaldehyde (PFA, pH 7.3) for 24 h and then immersed in 1% OsO_4_ for 1 h at room temperature [62]. Samples were then dehydrated through a series of solutions with ascending concentrations of ethanol and embedded in resin. Ultrathin sections were cut using an ultramicrotome (Leica), stained with 3% uranyl acetate and 1% lead citrate and then observed using a TEM (Hitachi H-7650, Japan).

### Haematoxylin and eosin staining

Tissues were fixed with 4% PFA for 24 h and then embedded in paraffin wax. Tissues were then consecutively sectioned into 5-μm-thick sections. The sections were stained with haematoxylin for 2 min and 1% eosin for 30 sec prior to observation. Ten sections from each tissue sample were analysed. The data were analysed using Image-Pro Plus 6.0.

### Immunohistochemistry and immunocytochemistry

Paraffin-embedded sections were incubated at 60°C for 2 h and then deparaffinised with xylene and rehydrated in a series of solutions containing descending concentrations of ethanol. For antigen retrieval, sections were pretreated with boiling citric acid buffer (10 mM, pH 6.0) for 15 min. After blocking with 10% normal donkey serum for 1 h at room temperature, sections were then incubated with the following primary antibodies overnight at 4°C: anti-LC3 antibodies (1:1,000 dilution) (Proteintech, Chicago, IL), anti-P62 antibodies (1:1,000, Proteintech, Chicago, IL), and anti-Atg7 antibodies (1:1,000, Proteintech, Chicago, IL). Sections were then incubated with FITC- or TRITC-conjugated secondary antibodies (Jackson ImmunoResearch, 1:100). Images were captured using a microscope attached to a charge-coupled device (CCD) camera (Leica, Germany).

### MDC staining

Cells were stained with MDC (Sigma, USA) and immediately analysed using fluorescence microscopy. Images were captured using a 380 nm excitation filter and a 525 nm emission filter.

### Plasma hormone measurements

Plasma testosterone and LH concentrations in breeding and nonbreeding males were detected using a radioimmunoassay kit (Xinfan Biotechnology, Shanghai, China), according to the manufacturer's instructions. Thirty-two samples were collected from 7 breeding males obtained from 7 colonies, and 20 samples were collected from 10 nonbreeding males obtained from 6 colonies. Culture media were submitted to different treatments for 24 h and then collected to determine the testosterone concentrations in Leydig cells from breeding and nonbreeding male NMRs. Testosterone production was detected using a radioimmunoassay kit (Sigma, USA) according to the manufacturer's instructions. All experiments were repeated three times. The testosterone concentration in the cultured medium was normalised to control for differences in cell numbers among different groups.

### Statistical analysis

Data are presented as the means±standard errors of the means (SEM), and significance was quantified by analysis of variance followed by Duncan's multiple comparison test. Western blot data were quantified by reporting the band density relative to β-actin and analysed using an unpaired *t*-test for independent samples. *P* < 0.05 was considered statistically significant.

### Abbreviations

NMR: naked mole-rat;SEM: standard error of the mean; CCD: charge-coupled device; PS: primary spermatocytes; SS: secondary spermatocytes.

## SUPPLEMENTARY MATERIALS FIGURES AND TABLES



## Abbreviations

NMR: naked mole-rat
SEM: standard error of the mean
CCD: charge-coupled device
PS: primary
