# Supplementary material for: Levels of Leydig cell autophagy regulate the fertility of male naked mole-rats
Source: Oncotarget. 2017 Oct 26;8(58):98677–90. doi: 10.18632/oncotarget.22088 (PMC5716759; doi:10.18632/oncotarget.22088)
Supplement: Supplementary file 1 [file oncotarget-08-98677-s001.pdf]

# Levels of Leydig cell autophagy regulate the fertility of male naked mole-rats

## SUPPLEMENTARY MATERIALS

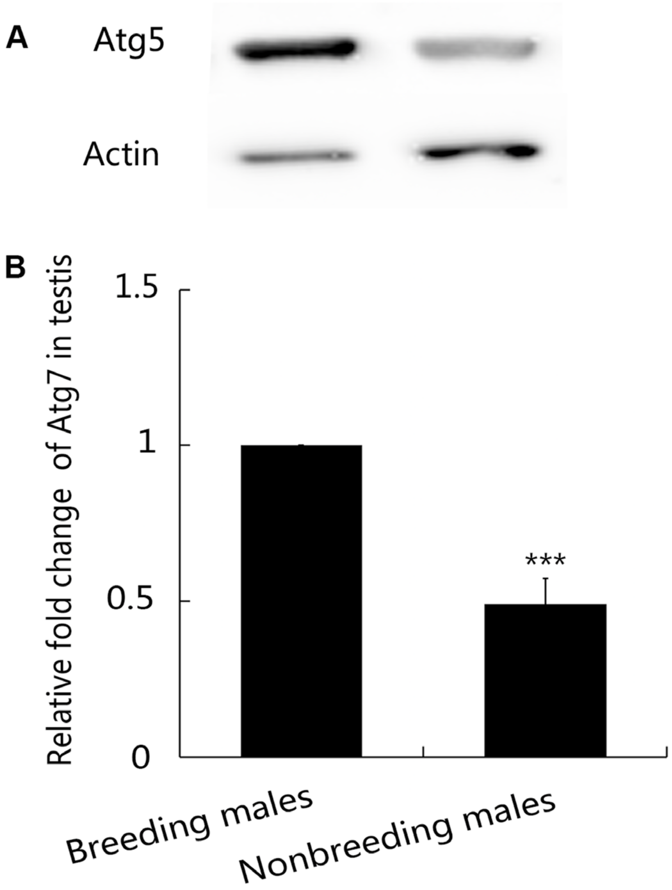


**Supplementary Figure 1: Atg5 levels in breeding and nonbreeding male NMRs.** (**A**) Western blot analysis of Atg5 levels in breeding and nonbreeding male NMRs. (**B**) Quantification of Atg5 levels in breeding and nonbreeding male NMRs ($P = 0.000879$) ($n = 6$ in each group). Data are presented as means ± SEM.

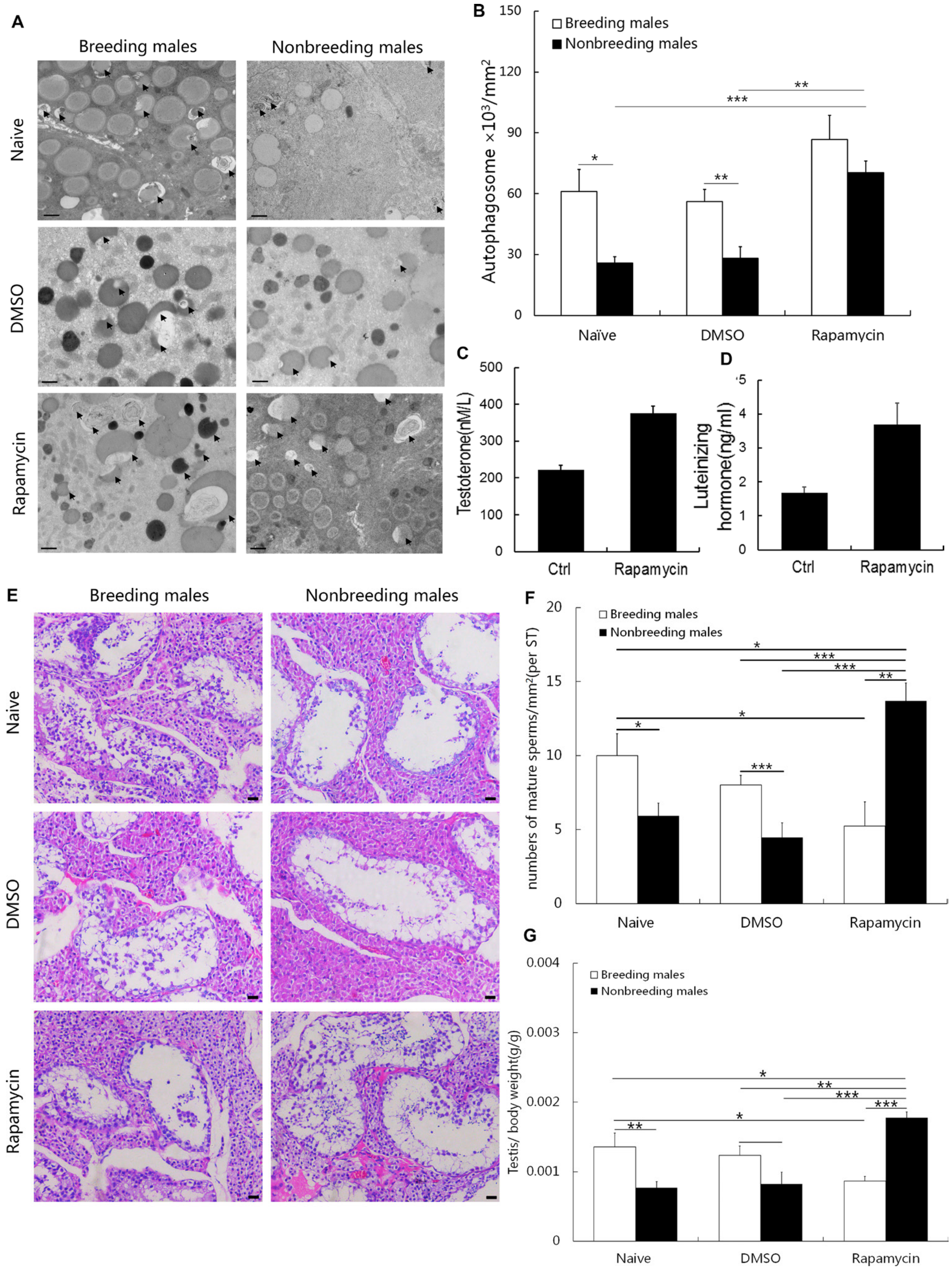


**Supplementary Figure 2: Rapamycin treatment restored the fertility of nonbreeding male NMRs.** (**A**) TEM was used to visualise Leydig cells from breeding and nonbreeding male NMRs. (**B**) Quantification of the autophagosomes (arrows) shown in (A) ($n = 6$ in each group). (**C** and **D**) Quantification of testosterone ($P = 0.0002910$) and LH ($P = 0.006257$) concentrations in nonbreeding male NMRs ($n = 6$ in each group), which were IP injected with the control or rapamycin. Data are presented as means±SEM. (**E**) HE staining was used to visualise sperm maturation status from breeding and nonbreeding male NMRs. (**F**) Quantification of the mature sperms shown in (E) ($n = 6$ in each group) (breeding males vs nonbreeding males in naive group, $P = 0.0146$, breeding males vs nonbreeding males in DMSO group, $P = 0.0007713$, breeding males in naive group vs in rapamycin group, $P = 0.0203$, breeding males vs nonbreeding males in rapamycin group, $P = 0.001995$, breeding males in naive group vs nonbreeding males in rapamycin group, $P = 0.02839$, breeding males in DMSO group vs nonbreeding males in rapamycin group, $P = 0.0001150$, nonbreeding males in DMSO group vs in rapamycin group, $P = 0.0005040$) (**G**) Quantification of testes weights from breeding and nonbreeding male NMRs under different treatment ($n = 6$ in each group)( breeding males vs nonbreeding males in naive group, $P = 0.009860$, breeding males vs nonbreeding males in DMSO group, $P = 0.02905$, breeding males in naive group vs in rapamycin group, $P = 0.02927$, breeding males vs nonbreeding males in rapamycin group, $P = 0.0001300$, nonbreeding males in naive group vs in rapamycin group, $P = 0.0001310$, breeding males in DMSO group vs nonbreeding males in rapamycin group, $P = 0.004080$, nonbreeding males in DMSO group vs in rapamycin group, $P = 0.009300$), which were IP injected with the control or rapamycin. Data are presented as means ± SEM.
